# Temperature and self-reported mental health in the United States

**DOI:** 10.1371/journal.pone.0230316

**Published:** 2020-03-25

**Authors:** Mengyao Li, Susana Ferreira, Travis A. Smith

**Affiliations:** Department of Agricultural and Applied Economics, University of Georgia, Athens, Georgia, United States of America; Columbia University, UNITED STATES

## Abstract

This study estimates the association between temperature and self-reported mental health. We match individual-level mental health data for over three million Americans between 1993 and 2010 to historical daily weather information. We exploit the random fluctuations in temperature over time within counties to identify its effect on a 30-day measure of self-reported mental health. Compared to the temperature range of 60–70°F, cooler days in the past month reduce the probability of reporting days of bad mental health while hotter days increase this probability. We also find a salience effect: cooler days have an immediate effect, whereas hotter days tend to matter most after about 10 days. Using our estimates, we calculate the willingness to pay to avoid an additional hot day in terms of its impact on self-reported mental health.

## Introduction

Average global temperature has increased by about 0.8˚C (1.4˚F) since global temperature record-keeping began in 1880, with two-thirds of this increase occurring in the last forty years. Eight of the top 10 warmest years on record for the United States of America (USA) contiguous 48 states have occurred since 1998, with 2016 reaching the greatest heat on record after three consecutive record-breaking years [1]. As one of the most important factors affected by climate change, human health is now recognized as a global research priority [2]. Heat stress triggers physiological responses in human bodies, from early signs of heat rash and muscle cramps to impacts on the central nervous system, circulatory system, and broad impacts on many organ systems [3,4]. High temperatures have been associated to negative birth outcomes [5,6], and to increased heat-related mortality, which would have been larger in the absence of air conditioning [7–9].

Besides studies on physical outcomes, an emerging literature has begun to pay attention to mental health outcomes by discovering increased reports of bad mental health [10,11], negative expressed sentiment [12,13], and suicide [14,10] as the temperature rises. Baylis et al. [12,13] employed billions of social media posts from millions of individuals using Facebook and Twitter, that were matched with gridded weather data and analyzed using a linear time-series cross-sectional model. They found a range of weather variables were associated with worsened expressions of sentiment: hot temperatures, precipitation, humidity, cloud cover, etc. Mullins and White [10] and Obradovich et al. [11] both used self-reported mental health survey data from the Centers for Disease Control and Prevention with varying study periods and empirical models. Both have found increased (risk of) reports of bad mental health days with more days falling in the hottest temperature bin in the last month. Burke et al. [14] tested the linkages between temperature and suicide rates in the USA and Mexico and found an increase of suicide rates of 0.7% in USA counties and 2.1% in Mexican municipalities with 1°C increase in monthly average temperature.

Our study analyzes the temperature and mental health relationship for the average adult in the USA. We exploit the exogenous year-to-year local variation in daily temperature to identify the short-run effect of temperature on mental health. The mental health question asks respondents about their mental health during the previous 30 days. Besides showing the unconditional relationship between temperature-day in bins and self-reported mental health as in Obradovich et al. [11] and Mullins and White [10], we further explore a saliency effect of persistent cold and hot days. Second, we estimate the effect of abnormal temperatures defined as deviations from the norm, where the norm is taken to be the average temperature over previous years or previous days. Third, we explore whether individuals have different expectations in response to temperature increases by season, and whether there is heterogeneity by climate region. Fourth, we compare the effects across people with and without frequent mental distress. Finally, we also contribute to the literature by estimating the economic costs of climate change by monetizing, as far as we know for the first time, the effect of temperature increases on individual mental health in two ways: (1) we adapt the health production model of Grossman [15] to estimate the willingness to pay (WTP) to maintain mental health well-being under rising temperatures assuming no adaptation cost (the lower bound); (2) we calculate the implicit marginal rate of substitution (MRS) between temperature and annual household income from a baseline regression model that controls for socio-economic and demographic information.

On the technical side, we empirically improve upon contemporaneous analysis in the following ways. First, even though we use the same surveillance data as Obradovich et al. [11] and Mullins and White [10], we limit the study period to 1993–2010, excluding those in 2011 and after as they are not directly comparable to those before 2011 due to changes in weighting methodology and cell phone sampling frames [16]. Second, since the self-reports of the mental health variable are count data with over sixty percent of respondents reporting zero days of bad mental health and right skewed, we apply a logistic model with a binary outcome variable. In contrast, ordinary least square methods [10] and linear probability models [11] can yield predictions outside the observable value range of the true data.

## Materials and methods

### Mental health and demographic information

Self-reports of mental health were obtained from the Behavioral Risk Factor Surveillance System (BRFSS), an ongoing state-based system of health surveys conducted annually under the auspices of the Centers for Disease Control and Prevention (CDC). Interviewers collect information on health risk behaviors, preventive health practices, and health care access primarily related to chronic disease and injury of the USA adult population (18 years and older) continuously through the year across all months evenly. Standardized core questionnaires with optional modules and state-added questions are conducted among states using Random Digit Dialing techniques on both landlines and cell phones. Weights are applied to each respondent to adjust for the bias of inequality in non-coverage and nonresponse among the population, and to force the total number of cases to equal population estimates for each geographic region. A mental health question became part of the core questionnaire in 1993. It asks: “Thinking about your mental health, which includes stress, depression, and problems with emotions, for how many days during the past 30 days was your mental health not good?” Answers range from 0 to 30 days. We define an individual as having self-reported mental health difficulties if they report 1 or more days of bad mental health.

**Fig 1:**
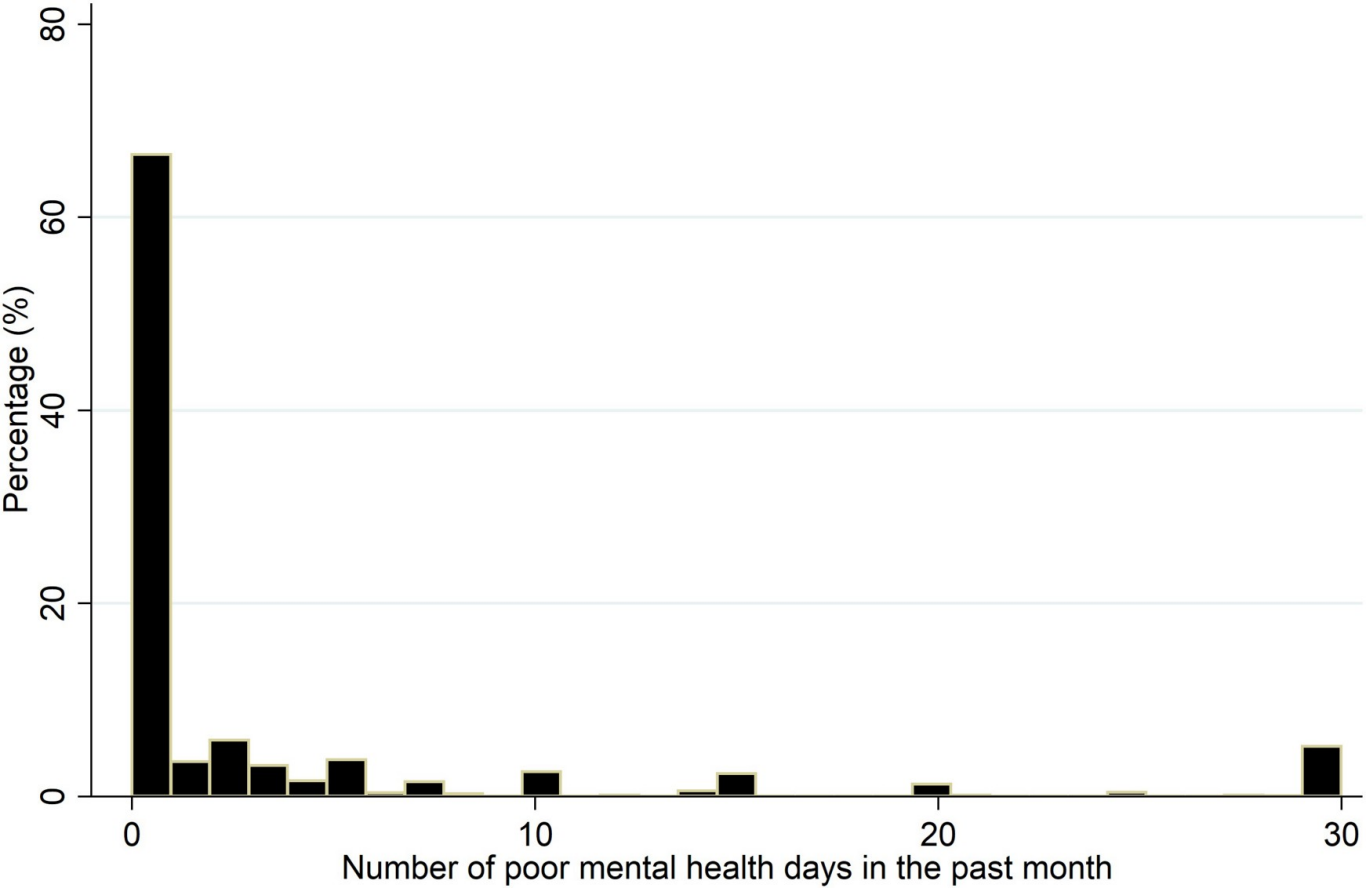
Distribution of bad mental health days. Data source for mental health: Behavioral Risk Factor Surveillance System (BRFSS) of the Centers for Disease Control and Prevention (CDC), 1993–2010 pooled cross-section. The distribution of bad mental health days corresponds to the question in the BRFSS survey regarding one's mental health condition (stress, depression, and problems with emotions) for the past month.

Fig 1 displays the histogram of the number of poor mental health days across the 48 contiguous USA states from 1993 to 2010 (Alaska, Hawaii, and the District of Columbia are not included). S1 Fig shows how bad mental health days are distributed across the USA. We limit our analysis to 2010 since data from 2011 and later are not directly comparable to those before 2011 due to changes in weighting methodology and cell phone sampling frame. Over 62% of the sample population responded with 0 days of bad mental health. We note that our measure is not based on professional diagnosis but rather on one’s own recall of the past 30 days. Therefore, there could be misreporting/recalling bias due to stigma/denial (i.e., the desire to under-report) and/or rounding (i.e., reporting “a couple of days” or “about one week”). However, this does not imply that self-reported mental health is not reliable. Hennessy et al. [17] use data from 1993 and found that “self-perceived health (including mental health) is a good proxy indicator for chronic disease conditions that have a heavy burden of symptoms”. Moreover, there is evidence that self-reported health data can be used reliably when medical claims and administrative data are unavailable [18]. More generally, indicators of self-reported subjective well-being have been found to be consistent, valid and reliable [19–21].

BRFSS also collects demographic information on age, sex, marital status, number of children, education, employment, annual household income, and whether the respondents have a healthcare plan. Race/ethnicity was not included as the BRFSS questionnaire options are not comparable across rounds. Table 1 displays a summary of the demographic information, with survey weight applied to reflect the census statistics. Among people who report at least one bad mental health day in the past month, about 35% have frequent mental distress (i.e. they report 14 or more days of bad mental health). Over 60% of the population have a partner, have some college experience, or are employed, respectively, and higher income groups have a larger population. The interview date and county Federal Information Processing Standards (FIPS) are used to merge responses from BRFSS to the temperature and other weather variables on the 30 days before and on the interview date.

**Table 1:** Weighted summary statistics of demographical information.

Continuous	Description	Mean (sd.)
Mental Health (# days)	Thinking about your mental health, which includes stress, depression, and problems with emotions, for how many days during the past 30 days was your mental health not good?	3.3 (7.3)
Temperature in bins	<20	12.6 (6.6)
(°F)	20–30	25.6 (2.8)
	30–40	35.3 (2.8)
	40–50	45.2 (2.9)
	50–60	55.0 (2.9)
	60–70	65.0 (2.9)
	70–80	74.8 (2.9)
	≥80	83.8 (3.4)
Precipitation (inch)	Average daily precipitation	0.1 (0.3)
Dewpoint temperature (°F)	Average daily dew point temperature	43.6 (17.8)
Sunlight (KJ/m^2^)	Average daily sunlight	16680.2 (7594.7)
**Categorical**	**Description**	**(%)**
Mental health difficulties	At least one day of bad mental health for the past 30 days	37.3
Frequent Mental Distress	Having ≥14 days of bad mental health for the past 30 days	13.2
Health Plan	No health plan	14.2
Elders	age≥65	15.0
Males	Male	49.6
Marital Status	Divorced	9.6
(Married omitted)	Widowed	5.9
	Separated	2.3
	Never married	18.6
	a member of an unmarried couple	3.7
Education	Elementary	3.6
(Never attended school omitted)	Some high school	6.8
	High school graduate	28.2
	Some college	27.5
	College graduates	33.6
Employment	Self-employed	8.4
(Employed for wages omitted)	Out of work >1 year	2.0
	Out of work <1 year	3.2
	Homemaker	7.3
	Student	4.1
	Retired	14.6
	Unable to work	3.9
Income	$10,000-$15,000	5.6
(<$10,000 omitted)	$15,000-$20,000	7.5
	$20,000-$25,000	9.3
	$25,000-$35,000	13.5
	$35,000-$50,000	16.8
	$50,000–75,000	17.5
	>$75,000	23.8
*Observations*	*N = 3*,*060*,*158*, *No*. *of Counties = 2*,*400*	

Data source for mental health: Behavioral Risk Factor Surveillance System (BRFSS) of the Centers for Disease Control and Prevention (CDC), 1993–2010 pooled cross-section; Data source for temperature, dewpoint temperature, and precipitation: PRISM Climate Group of Oregon State University; Data source for sunlight: CDC WONDER database. Survey weight was applied.

### Weather variables

**Fig 2:**
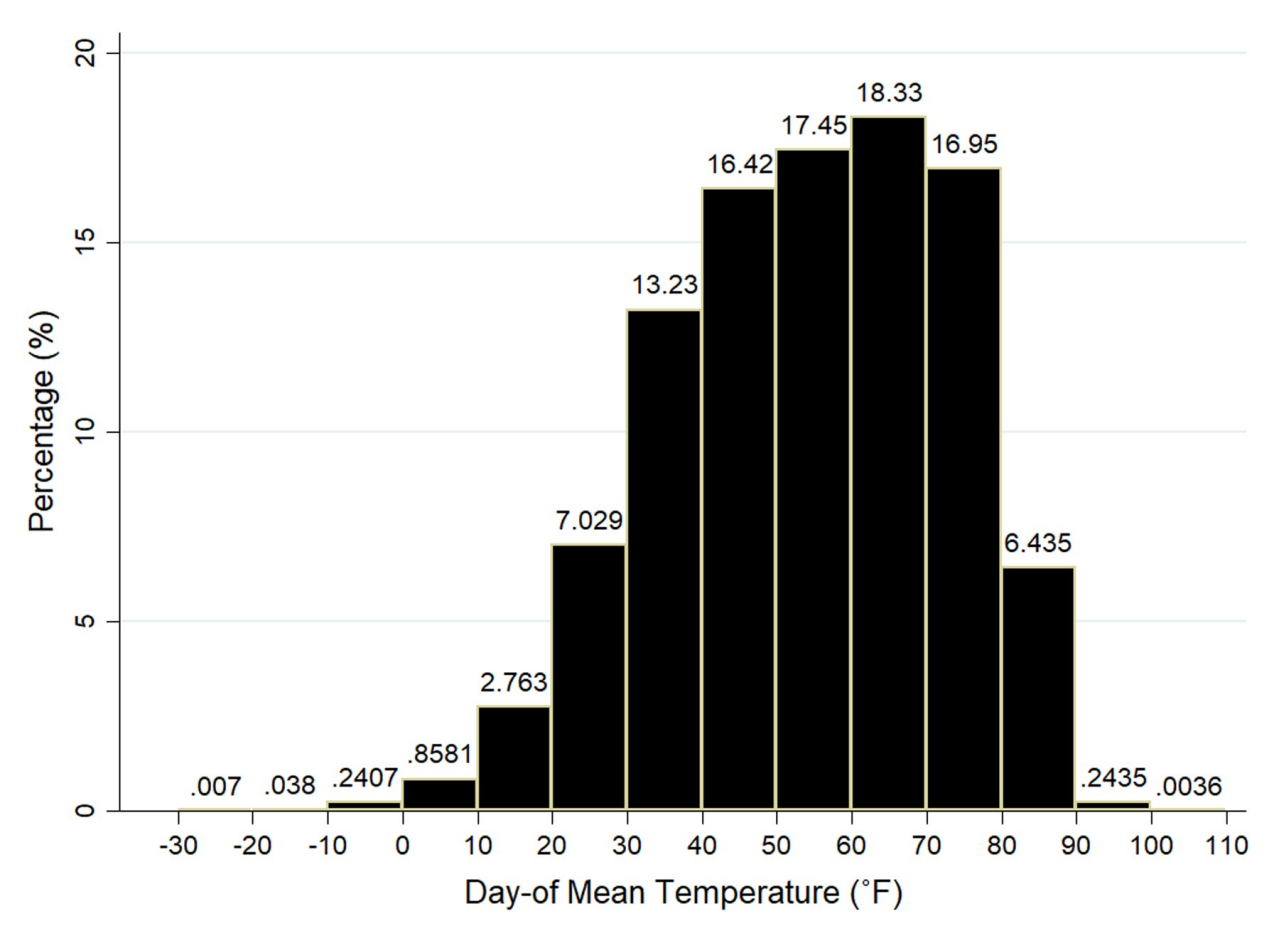
Distribution of interview date mean temperature. Data source for temperature: PRISM Climate Group of Oregon State University. Combined with BRFSS data for the period 1993–2010.

We obtain county-level daily temperature (maximum, minimum, mean, dewpoint) and precipitation from PRISM—Parameter-Elevation Regressions on Independent Slopes Model—developed by the Spatial Climate Analysis Service at Oregon State University for the National Oceanic and Atmospheric Administration given the centroid location of a county [22]. Daily averages are calculated based on hourly averages from the Integrated Surface Hourly network within PRISM that records data hourly. The regression-based PRISM model uses point data and other spatial datasets with a digital elevation model and an encoded spatial climate knowledge base to generate estimates of annual, monthly, daily, and event-based climatic elements [23]. It produces estimates of temperature and precipitation at county centroids by interpolating data from the 4×4-kilometer grid cells for the contiguous USA and each grid approximates the spatial unit by accounting for elevation, wind direction, rain shadows, and many other factors. PRISM is regarded as one of the most reliable interpolation procedures for climatic data on a small scale and is applied by NASA, the Weather Channel, and almost all professional weather services [24]. Auffhammer et al. [25] comment on the pitfalls of weather data and climate models used in economic analyses but confirm the reliability of the PRISM procedure in the USA where there are several thousand weather stations with daily records for many different weather indicators. The distribution of average temperature for the period under study is displayed in Fig 2. We then match temperature and precipitation from PRISM to the BRFSS dataset using date and county FIPS code.

Besides temperature and precipitation, we also collected daily sunlight duration data from the North America Land Data Assimilation System (NLDAS) maintained by the CDC WONDER [26]. The sunlight here reflects true sunlight exposure each day. It is important for mental health, and psychologists have used light as a treatment for patients with the seasonal affective disorder for decades [27–29].

### Empirical model

A key modeling issue in studies of heat stress is the existence of nonlinearities and threshold effects in temperature. Common empirical solutions include splines, threshold indicators, or temperature-day bins, as summarized by Deschenes [2]. Given that respondents to BRFSS questionnaires report the number of bad mental health days over the “past 30 days”, we use temperature-day bins by distributing the number of days in the previous 30 days to temperature bins based on the average daily temperature. As done elsewhere [7], we focus on the mean temperature in our study, noting that it will be correlated with maximum and minimum temperatures. The minimum temperature typically occurs in the middle of the night when people are asleep. The maximum temperature may be avoided by moving indoors [30], although this may not be possible for some types of jobs or activities.

We also note that there is a degree of subjectivity in the way people understand and report mental health: two individuals experiencing the same degree of stress or depression might report different experiences (or number of days), which makes interpersonal comparability of granular variation in self-reports difficult. Therefore, we create a binary variable for mental health difficulties, which takes the value of one for those who report one or more days of bad mental health. Thus, we do not explore the complete distribution of bad mental health days, but focus on the probability of self-reported mental health difficulties (i.e., reporting at least one bad mental health day).

We apply a logistic model:

$$MH_{ict}=\frac{\text{exp}\left(Z_{ict}\right)}{1+\text{exp}\left(Z_{ict}\right)}+\varepsilon_{ict},$$ (1)

where $Z_{ict}=\alpha +\sum_{k=1}^{7}\beta_{k}Temp_{ctk,d}+\gamma {\bar{W}}_{ct,d}+\delta X_{ict}+\theta_{Sm}+\theta_{c}+\theta_{dy}+\theta_{y}$.

*MH*_*ict*_ takes a value of 1 if a respondent *i* in county *c* on interview date *t* reported at least one bad mental health day during the past 30 days and 0 otherwise. $\sum_{k=1}^{7}\beta_{k}Temp_{ctk,d}$ captures the effect of temperature bin *k*, which ranges from less than 20°F to more than 80°F by 10°F-wide bins with 60–70°F bin, omitted as the base. The 60–70°F bin covers the commonly agreed upon temperature range where most humans feel comfortable, and it includes the temperature of 65°F, which is used to calculate heating degree days (HDD) and cooling degree days (CDD). The value in each bin represents the number of days in the past *d* days, from and including the interview date *t* in county *c*, for which the average daily temperature falls into that bin. This flexible specification of temperature does not require distributional assumptions on the temperature-mental health relationship. In the benchmark specification *d* = 30 so that it corresponds to the period of the mental health question (30 days), but we also present the results for alternative time horizons (*d* = 0, 7, 14 and 21 days), which allows us to explore the saliency temperature in more recent days, as well as cumulative effects of sustained very cold or hot days.

${\bar{W}}_{ct,d}$ is a vector of weather controls: precipitation, dewpoint temperature, and sunlight. Here, dewpoint temperature is the temperature at which relative humidity is 100%. At this point, additional water in air or a drop in temperature would result in condensation. This directly affects how comfortable the air feels and is used in the calculation for both relative and specific humidity. The National Weather Service [31] recommends dewpoint temperature as a better measure for air humidity than relative humidity. Barreca [32] found that specific humidity contributes the most to human well-being among other humidity measures. We calculated average dewpoint temperatures for the past *d* days from (and including) the interview date. *X*_*ict*_ includes the individual’s socio-economic and demographic information (age, age squared, Hispanic, marital status, number of children, sex, education, employment, household income, health plan).

As mentioned by Deschenes [2], identification of the linkage between extreme temperature and health is difficult due to nonlinearities reflected by complicated dynamic relationships, omitted variable bias, and secular or seasonality trends. Therefore, we further include a set of fixed effects: state-month dummies (*θ*_*Sm*_) account seasonal trends within each state (e.g., the within-year trend in Minnesota will be different than in Georgia); county dummies (*θ*_*c*_) control for time-invariant differences in unobserved mental health across very refined geographies; day-of-year dummies (*θ*_*dy*_) help to rule out within-year temporal differences that affect all USA residents; year dummies (*θ*_*y*_) control for the aggregate (macro) trends in self-reports of mental health (e.g., recessionary periods). Finally, we cluster standard errors at the county level.

## Results

Our results display the marginal effect (∂y/∂x) of the associated variables of interest. Table 2 shows marginal effects of temperature over the past month on reports of bad mental health. Each column represents a different regression with additional controls in order to show how confounders impact the relationship between temperature and mental health. For example, while the negative (positive) effect remains for the coldest (hottest) bin from columns (1) to (3), the marginal effect of other bins below 50°F change from positive to negative. When we add sunlight in column (4) and dewpoint temperature in column (5) the coefficients on all cooler bins are negative and those on hotter bins are positive and most of them are statistically significant at conventional levels. The final column is our preferred specification moving forward. S1 Table shows the full regression results (including individual covariates and weather variables) for this specification. As a robustness check, we also estimated a regression with the average temperature in levels and obtain a significant overall effect of average temperature for the past 30 days (result available upon request).

**Table 2:** Effect of temperature over the previous 30 days on the probability of self-reported mental health difficulties.

	(1)	(2)	(3)	(4)	(5)
Mean Temperature	Mental Health Difficulties	Mental Health Difficulties	Mental Health Difficulties	Mental Health Difficulties	Mental Health Difficulties
<20°F	-0.0096***	-0.0121***	-0.0059***	-0.0072***	-0.0083***
	(0.0017)	(0.0018)	(0.0019)	(0.0019)	(0.0032)
20–30°F	0.0075***	0.0048***	0.0020	0.0005	-0.0002
	(0.0016)	(0.0016)	(0.0016)	(0.0017)	(0.0025)
30–40°F	0.0048***	0.0027*	-0.0023*	-0.0038***	-0.0044**
	(0.0014)	(0.0014)	(0.0013)	(0.0014)	(0.0020)
40–50°F	0.0037***	0.0025**	-0.0010	-0.0022**	-0.0025**
	(0.0012)	(0.0011)	(0.0011)	(0.0010)	(0.0013)
50–60°F	-0.0002	-0.0010	-0.0010	-0.0017	-0.0019*
	(0.0011)	(0.0012)	(0.0011)	(0.0011)	(0.0011)
70–80°F	0.0047***	0.0054***	0.0016*	0.0018**	0.0020*
	(0.0009)	(0.0009)	(0.0009)	(0.0009)	(0.0011)
≥80°F	0.0066***	0.0079***	0.0025**	0.0029**	0.0032**
	(0.0011)	(0.0013)	(0.0012)	(0.0012)	(0.0015)
*N*	3,060,158	3,060,158	3,060,158	3,060,158	3,060,158
*No*. *of counties*	2,400	2,400	2,400	2,400	2,400
State-month	Y	Y	Y	Y	Y
County	Y	Y	Y	Y	Y
Day-of-year	-	Y	Y	Y	Y
Year	-	-	Y	Y	Y
Sunlight	-	-	-	Y	Y
Dewpoint temperature	-	-	-	-	Y

Columns (1) to (5) represents marginal effects from five separate logistic regressions. Standard errors are clustered at the county level in parentheses. State-month is the interaction of state and month dummies to control for local seasonality; county, day-of-year, and year are dummies that control for unobserved differences across counties, days of the year, and year. All regressions control for individual covariates (age and its squared, gender, income, marital status, number of children, education, employment, health plan) as well as average precipitation for the past 30 days. Both sunlight and dewpoint temperature are added as average on previous 30 days. Survey weights are applied. Bin of 60–70°F is omitted as the reference and the dotted line separates temperature cooler/hotter than the reference.

*** p<0.01,

** p<0.05,

* p<0.1.

In general, the probability of reporting mental health difficulties decreases with cooler days and increases with hotter days. Specifically, one additional day with an average temperature below 20°F leads to a 0.8% reduction in the probability of self-reported mental health difficulties for the past month; one additional day with average temperature above 80°F would lead to a 0.3% increase in that probability (compared to a change in the base bin). To help us understand the economic significance of this effect, we estimate the WTP for temperature-day changes below.

While the mental health question pertains to the past 30 days, it may be that more recent days are more salient on the minds of respondents. We explore this hypothesis in Table 3 by using alternative specifications with respect to recent temperatures. First, we examine the effect of the mean temperature of the interview date (or day-of temperature) in bins in column (1). We then estimate the effect of the number of days with average temperature in bins for the previous 1 week (*d* = 7 days), 2 weeks (*d* = 14 days), 3 weeks (*d* = 21 days), and 4 weeks (*d* = 30 days) in columns (2)-(5). All the regressions include the full set of controls (not reported). Across specifications, the negative (positive) effect on the cooler (hotter) bins is consistent, even for the interview day. The day-of temperature is not supposed to affect people’s recall of their past conditions—it should be the temperature in past days that matter. However, since temperatures are correlated over time, the effect on the day-of temperature could approximate that of temperature in previous days.

**Table 3:** Effect of temperature on the probability of self-reported mental health difficulties: Alternative specifications.

	(1)	(2)	(3)	(4)	(5)
	Day-of	Previous 7 Days	Previous 14 Days	Previous 21 Days	Previous 30 Days
<20°F	-0.0680**	-0.0237***	-0.0155***	-0.0112***	-0.0083***
	(0.0273)	(0.0079)	(0.0056)	(0.0041)	(0.0032)
20–30°F	-0.0101	-0.0026	-0.0038	-0.0014	-0.0002
	(0.0238)	(0.0053)	(0.0040)	(0.0030)	(0.0025)
30–40°F	-0.0097	-0.0066	-0.0075**	-0.0057**	-0.0044**
	(0.0191)	(0.0040)	(0.0030)	(0.0024)	(0.0020)
40–50°F	-0.0099	-0.0063**	-0.0052**	-0.0036**	-0.0025**
	(0.0135)	(0.0031)	(0.0024)	(0.0016)	(0.0013)
50–60°F	-0.0081	-0.0027	-0.0041**	-0.0026**	-0.0019*
	(0.0134)	(0.0024)	(0.0017)	(0.0013)	(0.0011)
70–80°F	0.0132	0.0054*	0.0040**	0.0035**	0.0020*
	(0.0126)	(0.0028)	(0.0020)	(0.0015)	(0.0011)
≥80°F	0.0040	0.0026	0.0048*	0.0044**	0.0032**
	(0.0183)	(0.0036)	(0.0027)	(0.0020)	(0.0015)
*N*	3,060,158	3,060,158	3,060,158	3,060,158	3,060,158
*No*. *of counties*	2,400	2,400	2,400	2,400	2,400

The dependent variable is a binary variable indicating whether the individual has self-reported mental health difficulties for all columns; column (1) looks at the day-of temperature in bins while columns (2)-(5) look at the temperature-day effect from previous 7 to 30 days. Standard errors are clustered at the county level in parentheses. State-month dummies are included to control for local seasonality; county, day-of-year, and year dummies are included to control for unobserved differences across counties, days of the year, and years. All regressions include individual covariates (age and its squared, gender, income, marital status, number of children, education, employment, health plan) as well as weather controls (precipitation, dew point temperature, sunlight). Temperature days in bin 60–70°F are omitted as the reference. Survey weight is applied.

*** p<0.01,

** p<0.05,

* p<0.1.

**Fig 3:**
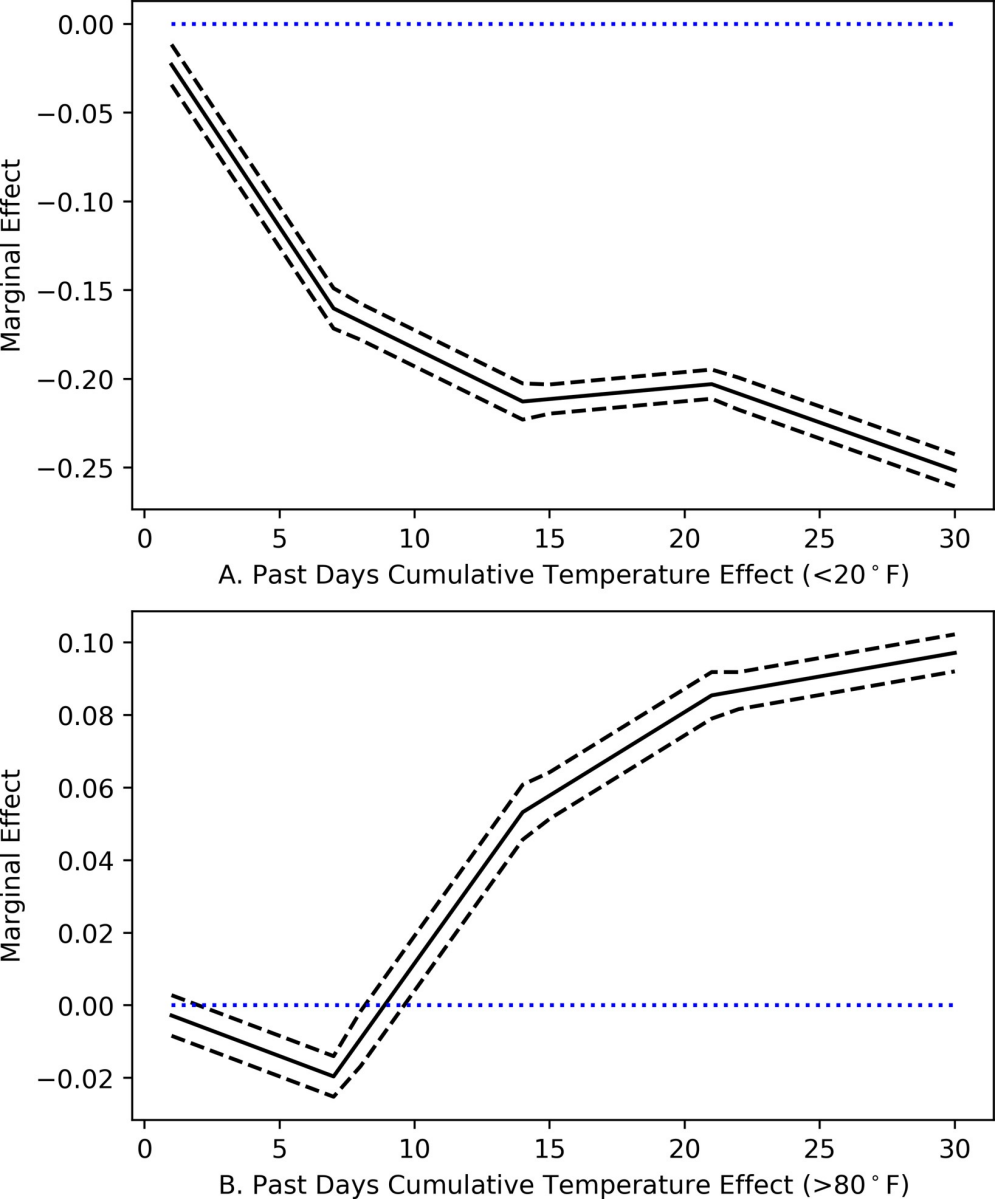
Effect of sustained very cold and hot days on the probability of self-reported mental health difficulties. Fig 3 is based on S2 Table. The slope is for the “cumulative effect” of the coldest (Fig 3A) and hottest (Fig 3B) bins for previous 1 to 4 weeks from the interview date that we have specified. The reference temperature bin for each week is 60–70°F. The dashed lines represent 90% confidence intervals with the blue line indicating non-effect.

The magnitude of the marginal effects for more recent weeks is larger, in general, as compared to more distant weeks. This could reflect the insights of Kahneman and Krueger [33] who suggest that people may upweight more recent experiences since they are reminisced more vividly. We further examine this pattern in S2 Table, where we look at the previous days’ marginal effect of temperature by week, conditional (Panel A) and unconditional (Panel B) on previous weeks. We focus on the temperature in the extremes, that is, bins with cold days with mean temperatures below 20°F and hot days with mean temperatures higher than 80°F. Fig 3 shows the conditional cumulative effect of sustained cold and hot days in each week for the past month based on Panel A of S2 Table. The slopes tell us how one additional day in the cold or hot bin is associated with the probability of self-reported mental health difficulties for that week after accounting for the previous week’s effects.

For cold days (Fig 3A), the effects of additional days in the <20°F bin on the probability of self-reported mental health difficulties are larger (in absolute value) in the first two weeks before the interview, suggesting that more recent cooler days are most salient. After the second week, the trend flattens out. In other words, sustained very cold days outside of two weeks do not work to increase mental health as compared to more recent cold days. For sustained hotter days (Fig 3B), no immediate effect is observed, and it appears that the probability of reporting mental health difficulties begins to rise after about 10 days before flattening out over the most distant week. Kahneman and Krueger [33] describe the retrospective evaluation of experiences as a weighted sum of moment-by-moment experiences. Depending on the context, individuals may place higher weights on more recent experiences (as is the case with cooler days), or they may place higher weights on the duration of events (as in the case of hotter days). In general, sustained cold days help to reduce the probability of reporting bad mental health days while sustained hot days tend to increase such a probability. Similar trend has also been revealed by suicide rates in the USA, which are lowest during the winter months and highest in the spring and summer [34].

We further explore respondents’ reactions to the day-of temperature by looking at its deviation from the local norm in Table 4. One hypothesis is that it could be the deviations in day-of temperature from what is expected or “normal” that is associated with people’s report of mental health, rather than the temperature itself. We find no such evidence.

**Table 4:** Effect of day-of temperature deviations on the probability of self-reported mental health difficulties.

	(1)	(2)	(3)
	Deviation from previous 10-year average	Deviation from previous 30-day average	Deviation from previous 7-day average
<-10°F	-0.0151	0.0141	0.0167
	(0.0141)	(0.0126)	(0.0102)
[-10, -5) °F	-0.0060	0.0100	0.0030
	(0.0087)	(0.0103)	(0.0079)
[-5, 0) °F	-0.0031	0.0039	0.0029
	(0.0070)	(0.0063)	(0.0068)
[5,10) °F	0.0031	-0.0099	-0.0057
	(0.0077)	(0.0081)	(0.0087)
≥10°F	-0.0015	-0.0011	-0.0006
	(0.0131)	(0.0124)	(0.0122)
*N*	3,060,158	3,060,158	3,060,158
*No. of counties*	2,400	2,400	2,400

The dependent variable is a binary variable indicating whether the individual has self-reported mental health difficulties; Day-of mean temperature is used to calculate temperature deviations in columns (1)-(3). Standard errors are clustered at the county level in parentheses. State-month dummies are included to control for local seasonality; county, day-of-year, and year dummies are included to control for unobserved differences across counties, day of the years, and year. All regressions include individual covariates (age and its squared, gender, income, marital status, number of children, education, employment, health plan) as well as weather controls (precipitation, dew point temperature, sunlight). Temperature deviations that fall in bin 0–5°F are omitted as the reference. Survey weight is applied. *** p<0.01, ** p<0.05, * p<0.1.

In column (1) of Table 4, we calculate the deviation of the day-of mean temperature from the average temperature for the same day of the year as the interview date, along with one week before and after that date, of each of the previous 10 years. In columns (2) and (3), we examine the deviation from the average temperature for the previous 30 days and 7 days, respectively. We assign the temperature deviation into six bins from less than -10°F to more than 10°F by 5°F-wide bins, with the bin of 0–5°F omitted as the base. Except for the first coefficient in column 3 at the ten percent level, no other coefficients in Table 4 are significant, suggesting that temperature deviations on the interview date are not associated with the probability of self-reporting mental health difficulties.

### Robustness by season and region

The results presented so far represent average effects across the USA from regressions that control for seasonality (by including state-month dummies). To examine seasonal and spatial patterns more closely, we also check the marginal effect by season and region in Tables 5 and 6. Since the average temperature is different by season, comparing temperature-day in bins to the same baseline of 60–70°F might mask some important differences. To make comparisons less complicated, we analyze the average temperature for the past 30 days in levels. Results are shown in Table 5, with panel A displaying the marginal effect of temperature in the previous 30 days by season based on the interview date and panel B deleting observations in the first month of a season to ensure that the previous 30-day temperature still falls in the same season as the interview date. For the same unit of increase in average temperature, the probability of having self-reported mental health difficulties for USA adults is significantly larger in Summer and Spring in both panels. Findings here are consistent with the literature that shows higher suicide rates in seasons other than Winter [34] and a higher probability of mental health issues in Spring and Summer [11].

**Table 5:** Effect of temperature over previous 30 days on the probability of self-reported mental health difficulties: By season.

	Winter	Spring	Summer	Fall
	(1)	(2)	(3)	(4)
**A. By 3-month in a Season**				
Past 30-day average temperature (°F)	0.0030	0.0093**	0.0129***	-0.0010
	(0.0039)	(0.0039)	(0.0048)	(0.0024)
Temperature (°F)				
Average (s.d.)	39.2 (15.0)	54.8 (14.0)	74.7 (8.5)	59.9 (13.4)
Minimum	-28.3	-13.7	27.5	-8.2
Maximum	80.9	94.8	102.8	99.5
Interview Months	Dec, Jan, Feb	Mar, Apr, May	Jun, Jul, Aug	Sep, Oct, Nov
*No*. *of Counties*	2,392	2,392	2,396	2,392
*N*	739,138	779,834	761,498	774,985
**B. By 2-month in a Season**	(5)	(6)	(7)	(8)
Past 30-day average temperature (°F)	0.0003	0.0091*	0.0106**	0.0025
	(0.0047)	(0.0049)	(0.0048)	(0.0034)
Temperature (°F)				
Average (s.d.)	38.4 (15.4)	59.3 (11.7)	76.2 (7.7)	55.2 (12.5)
Minimum	-28.3	6.2	35.6	-8.2
Maximum	80.9	94.8	102.8	88.9
Interview Months	Jan, Feb	Apr, May	Jul, Aug	Oct, Nov
*No*. *of Counties*	2,386	2,382	2,391	2,387
*N*	485,205	517,384	508,684	526,762

Regressions follow the baseline regression in Table 2 column (5) but replace temperature-day bins with the average past 30-day temperature. Columns (1)-(4) in panel A include all interview months within the corresponding season while columns (5)-(8) in panel B exclude the first month of that season to ensure the previous 30-day temperature are within the same season as the interview date.

*** p<0.01,

** p<0.05,

* p<0.1.

**Table 6:** Effect of temperature over previous 30 days on the probability of self-reported mental health difficulties: By climate region.

	(1)	(2)	(3)	(4)	(5)	(6)	(7)	(8)
	Hot	Mild Hot	Mild Cold	Cold	Hot	Mild Hot	Mild Cold	Cold
	lat≤35°	35°<lat≤40°	40°<lat≤43°	lat>43°	Temp<47.6°F	47.6°F< Temp≤54.9°F	54.9°F< Temp≤62.4°F	Temp≥62.4°F
<20°F	-0.0103	-0.0108**	-0.0030	-0.0016	0.2246**	0.0136	-0.0064	-0.0019
	(0.0303)	(0.0046)	(0.0054)	(0.0051)	(0.1129)	(0.0086)	(0.0043)	(0.0012)
20–30°F	-0.0039	-0.0040	0.0061	0.0020	0.0038	-0.0014	0.0037	-0.0015
	(0.0086)	(0.0039)	(0.0047)	(0.0045)	(0.0139)	(0.0061)	(0.0035)	(0.0010)
30–40°F	-0.0073*	-0.0080**	0.0019	-0.0025	-0.0095	-0.0044	-0.0019	-0.0014
	(0.0044)	(0.0036)	(0.0038)	(0.0035)	(0.0059)	(0.0049)	(0.0031)	(0.0009)
40–50°F	-0.0031	-0.0026	-0.0022	-0.0009	-0.0027	-0.0015	-0.0005	-0.0015**
	(0.0022)	(0.0026)	(0.0031)	(0.0027)	(0.0023)	(0.0032)	(0.0025)	(0.0006)
50–60°F	-0.0009	-0.0041*	0.0018	-0.0011	-0.0015	-0.0030	0.0002	-0.0007
	(0.0015)	(0.0024)	(0.0027)	(0.0024)	(0.0020)	(0.0024)	(0.0022)	(0.0005)
70–80°F	0.0036*	0.0001	0.0027	0.0033	0.0047***	-0.0000	0.0032*	0.0003
	(0.0019)	(0.0016)	(0.0021)	(0.0025)	(0.0016)	(0.0022)	(0.0018)	(0.0005)
≥80°F	0.0047**	0.0043**	-0.0045	0.0071	0.0057***	0.0013	0.0035	0.0005
	(0.0023)	(0.0021)	(0.0036)	(0.0068)	(0.0019)	(0.0027)	(0.0035)	(0.0023)
*N*	620,294	861,239	894,323	680,083	497,339	698,296	1,228,185	632,168
*No*. *of Counties*	638	870	468	424	474	719	728	479
# days/yr average temperature>70°F	161	97	61	34	173	114	67	33

Regressions follow Column (5) in Table 2; The climate regions are divided based on latitude of the centroid location of the county for columns (1) to (4), and by the average daily temperature over the study period for columns (5) to (8); The number of days above 70°F each year is also displayed.

*** p<0.01,

** p<0.05,

* p<0.1.

Table 6 explores the effect of temperature by climate regions (Hot, Mild Hot, Mild Cold, and Cold) based on either the latitude of the counties’ centroids (regardless of county altitude) or the average daily temperature over the study period (considers counties in higher altitude at lower latitude): when ranking latitude from south to north or average daily temperature from high to low, Hot and Cold regions are defined as the first and last 20% of all counties; for the rest 60% in the middle, they are split evenly by assigning the former 30% as Mild Hot regions and the other 30% as Mild Cold regions. We keep less percentage of counties for the Hot and Cold regions as the distribution of temperature is bell-shaped (Fig 2) and counties with daily temperatures at the extreme are less relative to counties with mild daily temperatures. The number of hot days in the Hot region is about 5 times that in the Cold region. We notice that additional hot days significantly increase the probability of self-reported mental health difficulties for the hot region, regardless of how the region is defined. It does not seem that people adapt to hotter climates. Such an increase in hot temperature days is not associated with the probability of self-reported mental health difficulties as much in Mild Cold or Cold regions, revealing more endurance of heat for people living in cooler areas where the average number of hot temperature days (>70°F) in a year is less. The effect of additional cooler days among regions is minimal, and it generally helps to reduce the probability of self-reported mental health difficulties.

### Frequent mental distress

**Fig 4:**
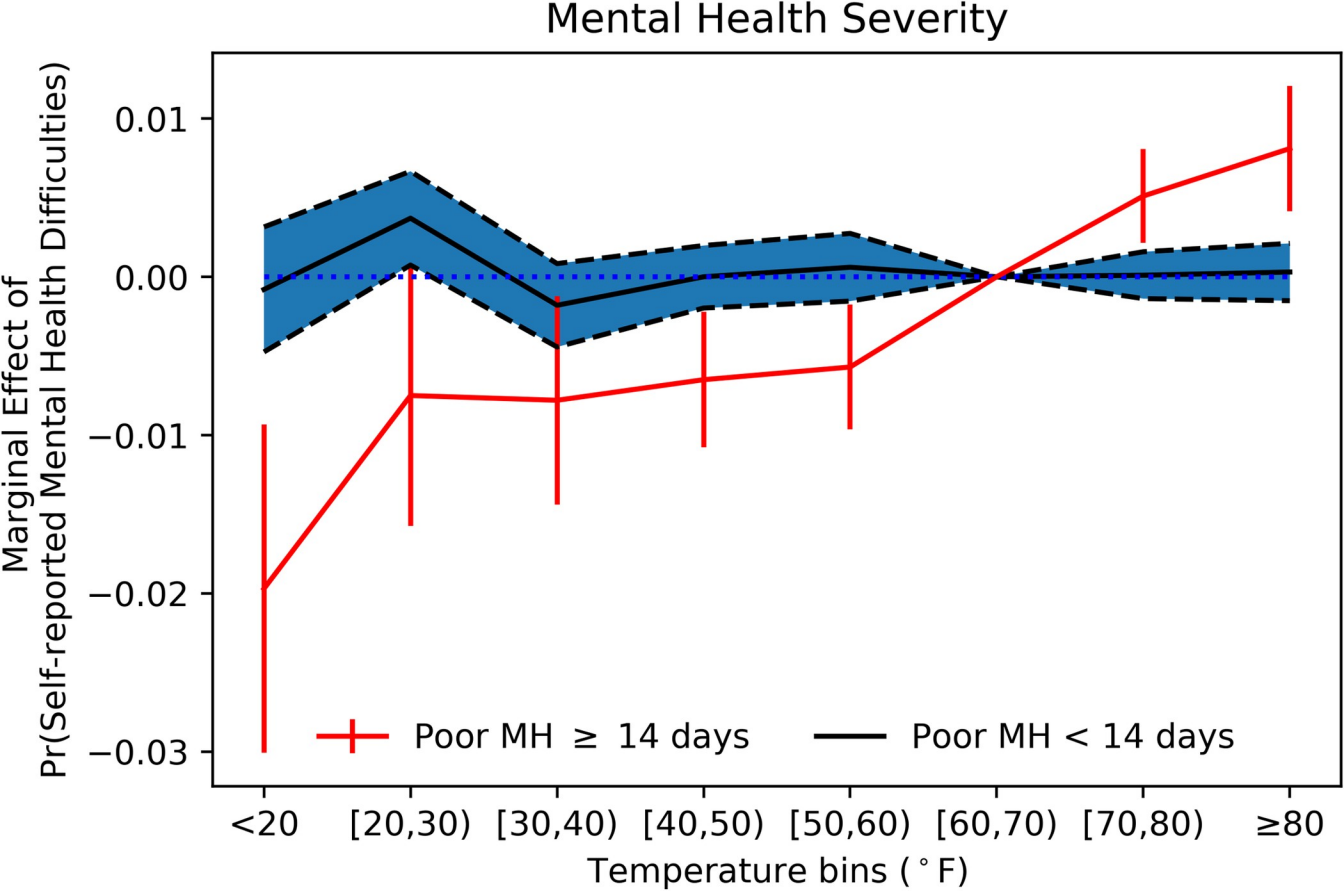
Robustness check by frequent mental distress. The sample is divided based on whether respondents have frequent mental distress (report more than 14 days of bad mental health for the past month). All individual covariates, weather, and location/time controls are included with survey weight applied and 90% confidence intervals displayed.

We further explore how temperature affects the probability of self-reported frequent mental distress (FMD) in Fig 4, which the CDC defines as reporting at least 14 days of bad mental health in the previous 30 days [35].

The effect of temperature on the probability of reporting FMD is significantly different from reporting between 1 and 13 days of poor mental health. The point estimates at extreme temperatures are larger in absolute value for the subsample of those experiencing FMD. The estimates of the marginal effect of temperature-day on those with and without FMD in Fig 4 are displayed in S3 Table. As an additional robustness check, we added one more column to S3 Table which focuses on those reporting 30 days of bad mental health. In this case, the marginal effect on temperature-day is even more salient.

### Analysis of willingness to pay

To help us understand the economic significance of the effects of temperature change on the probability of self-reported mental health difficulties, we derive two practical expressions for estimating the health-related welfare effect of temperature changes: the first one comes from a mental health production function and the second one is the implicit marginal rate of substitution between temperature and annual household income in Eq (1).

#### Mental health production

We adapt Deschenes and Greenstone's [7] analysis of temperature and mortality to mental health. The conceptual framework is originally derived from the Becker-Grossman static model of health production [15] where health is both demanded and produced by consumers. We assume individuals maximize utility over the consumption of a single numeraire good *X*_*c*_ and mental health *MU*: *U* = *U* (*Xc*, *MH*). We further assume that mental health is produced/generated with a vector of mental health-maintaining market goods *Xm* with a relative price p and is further dependent upon temperature *T*: *MH* = *f* (*Xm*, *T*), where *Xm* is assumed to be a function of *T*, so that $\frac{\text{dMH}}{\text{dT}}=\frac{\partial \text{f}}{\partial \text{Xm}}\frac{\partial \text{Xm}}{\partial \text{T}}+\frac{\partial \text{f}}{\partial \text{T}}$ and the purchase of market goods (*Xc* and *Xm*) is constrained by income *I*. After some manipulation (Check Deschenes and Greenstone [7] for a detailed derivation), we can obtain an expression for the change in income necessary to keep utility constant when *T* changes, or the Willingness to Pay (WTP) for changes of *T*:

$$\frac{\text{dI}}{\text{dT}}{|}_{du=0}=-\frac{\text{df}}{\text{dT}}\frac{\partial \text{U}}{\partial \text{f}}/\lambda +\text{p}\frac{\partial \text{Xm}}{\partial \text{T}}.$$ (2)

Here, $\frac{\partial U}{\partial f}/\lambda$ is the dollar value of the disutility of a change in mental health, or in other words, the WTP to maintain one’s mental health condition. Morey, Thacher and Craighead [36] give the most updated estimates of absolute WTP for eliminating depression using a discrete-choice random-utility model among patients with major depressive disorder (MDD). The original WTP for a representative individual to eliminate MDD with no side effects in the paper before adjusting for inflation is $686 in 2007 dollars. Using the consumer price index in 2007 and 2017 (207.3 and 245.1) [37], we calculated the value in 2017 to be $686×245.1/207.3 = $811. The monthly expected WTP to eliminate MDD with no side effects is about $811 given inflation.

We use the above value as an approximation for $\frac{\partial U}{\partial f}/\lambda$ and note two caveats. First, depression is only one symptom of mental unhealthiness: others mentioned in the BRFSS questionnaire include stress and problems with emotions. Second, it is not clear whether eliminating MDD is as expensive as eliminating any bad mental health day (i.e., returns to scale with respect to temperature in the production of mental health could be increasing, decreasing or constant at different stages of production). Thus, we consider our evaluation for $\frac{\partial U}{\partial f}/\lambda$ to be a rough estimate.

$\frac{\text{df}}{\text{dT}}$ is the change in the risk of having bad mental health given an additional day in extreme for the past month, which is the total derivative of the mental health function with respect to temperature and could be obtained through regressing the probability of self-reported mental health difficulties on temperature-day in bin without controlling for *Xm*. Under the logistic setting of our empirical model, $\frac{\text{df}}{\text{dT}}$ can be obtained from the marginal effects reported in the last column of Table 2. For calculations, we only focus on the extreme temperature bins of <20°F and ≥80°F given their larger and salient effect for the past month.

$\frac{\partial \text{Xm}}{\partial \text{T}}$ represents how the demand for market goods that produce mental health change with temperature. Adaptation to temperature changes with regards to mental health could involve an increasing reliance on services such as counseling or medication. Mullins and White [10] examined the adaptation to temperature in the context of emergency room visits in California and suicide rates by county. They find no evidence of adaptation even after accounting for a broad list of possible moderators: air conditioning, mental health parity laws, mental health professional shortage areas, substance abuse treatment centers, county median income.

If we conservatively assume the effect of temperature variations on mental health maintaining products to be zero ($\frac{\partial \text{Xm}}{\partial \text{T}}=0$), the lower-bound estimation of WTP to maintain good mental health for a month would be estimated $811* $\frac{\text{df}}{\text{dT}}$ in 2017 dollars. Table 7 presents the previous 30-day extreme temperature effect on mental health and the associated WTP. On average, people would be willing to pay $6.7 for one more day in the past month with temperature below 20°F compared to the human comfortable range, or $2.6 for one fewer day with temperature higher than 80°F.

**Table 7:** Willingness to pay for temperature-day changes.

	df/dT (SD)	WTP—MH function (95% CI)	WTP—MRS income (95% CI)
**Previous 30 Days**			
<20°F	-0.0083***	$6.7	$10.7
	(0.0032)	($1.6-$11.8)	($2.7-$17.6)
≥80°F	0.0032**	$2.6	$4.6
	(0.0015)	($0.2-$5.0)	($0.2-$8.9)

WTP = $-\frac{\text{df}}{\text{dT}}\frac{\partial \text{U}}{\partial \text{f}}/\lambda +\text{p}\frac{\partial \text{Xm}}{\partial \text{T}}$, where $\frac{\partial U}{\partial f}/\lambda$ is assumed to be $811 in 2017 dollars, and $\frac{\partial \text{Xm}}{\partial \text{T}}$ = 0 [36,10]. Only extreme temperature-day effect of <20°F and ≥80°F for the previous 30 days are presented. df/dT is the marginal effect as displayed in S1 Table. The implicit MRS is the average marginal rate of substitution between temperature and annual household income; confidence intervals are given by the Delta method.

*** p<0.01,

** p<0.05,

* p<0.1.

#### Marginal rate of substitution

Another way for us to estimate WTP is to look at the implicit marginal rate of substitution (MRS) between temperature and the log of annual household income that keeps the probability of changes in mental health constant [38–40]. When totally differentiating the empirical model and holding the probability of changes in mental health constant, the implicit MRS between temperature and annual household income equals $-\bar{Inc}\left(\frac{\partial \text{P}\left(\text{z}\right)}{\partial Tbin}/\frac{\partial \text{P}\left(\text{z}\right)}{\partial \text{ln}\left(Inc\right)}\right)$, where $\bar{Inc}$ is the annual household income adjusted by the consumer price index in 2017 ($58,820), $\frac{\partial \text{P}\left(\text{z}\right)}{\partial Tbin}$ is the marginal effect of an additional day in extreme on the risk of self-reported mental health difficulties for the past month, and $\frac{\partial \text{P}\left(\text{z}\right)}{\partial \text{ln}\left(\text{Inc}\right)}$ is the marginal effect of log of income (check Marginal Effect Calculation in Supporting Information for the full derivation and refer to S1 Table for the values on marginal effects). Taking the hottest temperature bin effect for an example, $\frac{\partial \text{P}\left(\text{z}\right)}{\partial Tbin}$ would be 0.0032 and $\frac{\partial \text{P}\left(\text{z}\right)}{\partial \text{ln}\left(\text{Inc}\right)}$ would be -0.1130, thus an additional day in the previous month with average temperature above 80°F (compared to the base bin) increases the probability of self-reported mental health difficulties by an amount worth $1,666 per year per person, which sums the dollar value of changes in bad mental health for each day of the year with an additional day in extreme in its previous month. Thus, we divide it by 365 days to obtain the dollar value of the change for any given day in a year, which amounts to $4.6 per day per person (2.8% of income). Similarly, the WTP for one more day with temperature below 20°F for the past month amounts to $10.7 per day (6.2% of income).

## Conclusions and discussions

Mental health has been gaining attention among world leaders in recent years. The promotion of mental health has—for the first time—been included in the United Nation Sustainable Development Agenda under goal number 3 (Good Health and Well-being) to be reached by 2030 [41]. In a rapidly warming world, temperature increases pose a challenge to achieving that goal. Our study attempts to gauge the magnitude of that challenge by quantifying the effect of temperature on self-reported mental health.

Overall, compared to the human comfortable temperature range of 60–70°F, additional cold days in the past month reduce the probability of self-reported mental health difficulties while additional hot days increase such probability. The effects are more marked for the probability of self-reported frequent mental distress. Our finding that additional hot days are correlated with increased self-reported mental distress is consistent with recent literature that analyzes the relationship between temperature and alternative measures of mental health [10, 11, 13]. The effect of cold days is a little more controversial: Obradovich et al. [11] do not find a statistically significant effect of cooler temperatures on self-reported mental health; Baylis et al. [13] shows that the expression of negative emotions on Facebook and Twitter increase in cooler days; while, consistent with our results, Mullins and White [10] show a beneficial effect of cooler temperature bins on mental health related to emergency department visits and suicide.

One potential mechanism of the beneficial mental effect of cooler temperatures could be due to the better quality of sleep. Mullins and White [10] show that with increased cooler days in the past month, the number of nights with poor sleep decreases and the minutes slept in the previous night increases. This confirms previous findings by Obradovich et al. [42] that cooler nighttime temperature anomalies significantly reduce nights of poor sleep and hotter temperature anomalies lead to significant increases. The relationship between poor sleep and bad mental health has been revealed by previous studies analyzing the role of factors other than temperature that affect both sleep and suicide attempts/rates [43,44].

We also find that people react to sustained cooler and hotter days differently: sustained cooler days do not contribute to increased mental distress over and beyond the most recent cold days, whereas the opposite is true for sustained hotter days. To the best of our knowledge, this is the first attempt to explore such differences. Moreover, we find no relationship between deviations in the day-of temperature from the local normal climate and people's recall of their past-month mental health condition. Further, a closer examination by season indicates that the adverse effect of higher temperature is worse in the Summer. Finally, examining heterogeneity by region reveals that warmer regions are particularly affected by additional hot days, while cooler regions are less affected by temperature in general. The estimated economic cost, measured in willingness-to-pay (WTP) to avoid an additional hot day in the past month, ranges from $2.6 to $4.6 per day.

The study is not free from limitations. We realize that the specific wording of survey questions is a key component of how respondents perceive the meaning of the question. The mental health question in the BRFSS includes specific examples: stress, depression, and problems with emotions. However, the way the question is framed might influence respondents to narrow or broaden the meaning of the question, which is true for most empirical analysis using surveys related to self-assessment.

The cross-sectional nature of the design also makes it hard to determine causality between temperature and bad mental health days. Clearly, we cannot randomly assign individuals to locations with different temperatures to identify a clear causal impact of temperature on self-reported mental health. Thus, we use econometric techniques to isolate the effect of temperature from other confounders. The validity of this paper’s empirical strategy rests crucially on the assumption that estimation of Eq (1) will produce unbiased estimates of the *β*_*k*_ vector. By conditioning on county and state-by-season fixed effects, *β*_*k*_ is identified from county-specific deviations in weather from the within-county averages after controlling for shocks common to all counties in a state in a season. Since weather fluctuations are unpredictable, it is reasonable to presume that this variation is orthogonal to unobserved determinants of health outcomes [7], including self-reported mental health difficulties, which gives us unbiased estimation of the *β*_*k*_ vector. However, there is indeed a source of bias from the nonlinear modeling of logit regression using the maximum likelihood estimation method, which diminishes as the sample size increases (in the case of clustered standard error, with the number of counties). Therefore, we do not claim a causal impact from temperature on self-reported mental health.

Moreover, although our findings benefit from a rich individual-level mental health dataset matched with high resolution temperature data varying daily by county, the data could not be matched to information on individual access to market or public goods that produce mental health, at either individual or community level. Yardley, Sigal and Kenny [45] point out that community level factors (e.g. social isolation, ethnicity, socioeconomic status, and the neighborhood social environment) may influence the impact of heat on human health. Due to data limitations, a community analysis was not achievable. It would be interesting for future studies to shed light on how community level factors mediate the effects of climate change on individual mental health and design policies accordingly.

## Supporting information

S1 Fig Geographic distribution of bad mental health days. The county-level average number of self-reported bad mental health days is smoothed for the period 1993–2010. A darker color signifies more self-reports of bad mental health. The southeastern area near the Gulf of Mexico and areas in Appalachia have higher counts of bad mental days as compared to the rest of the country.
(DOCX)

S1 Table Full regression of baseline specification. Marginal effects from logistic regression are displayed. Standard errors are clustered at the county level in parentheses. Controls for State-month, county, day-of-year, and year dummies are also included but not displayed to save space. Survey weight is applied. *** p<0.01, ** p<0.05, * p<0.1.
(DOCX)

S2 Table Effect of temperature from previous days by week: Conditional and unconditional. Columns in Panel A look at the effect of temperature for previous 7 to 30 days accordingly by week, conditional on previous weeks; columns in Panel B look at the temperature effect for previous weeks by week, unconditional on previous weeks. Each column follows the baseline specification in Eq (1). Survey weight is applied, and marginal effects from logistic regressions are displayed. *** p<0.01, ** p<0.05, * p<0.1.
(DOCX)

S3 Table Effect of temperature on people with frequent mental distress (FMD). The regression results of marginal effects of the first two columns are supplement for Fig 4; *** p<0.01, ** p<0.05, * p<0.1.
(DOCX)

S1 Text Marginal effect calculation. Derive the marginal effect from the logistic regression.
(DOCX)
