# Supplementary material for: Temperature and self-reported mental health in the United States
Source: PLoS One. 2020 Mar 25;15(3):e0230316. doi: 10.1371/journal.pone.0230316 (PMC7094821; doi:10.1371/journal.pone.0230316)
Supplement: S1 Fig — The county-level average number of self-reported bad mental health days is smoothed for the period 1993–2010. A darker color signifies more self-reports of bad mental health. The southeastern area near the Gulf of Mexico and areas in Appalachia have higher counts of bad mental days as compared to the rest of the country. (DOCX) [file pone.0230316.s001.docx]

**S1 Fig. Geographic distribution of bad mental health days**


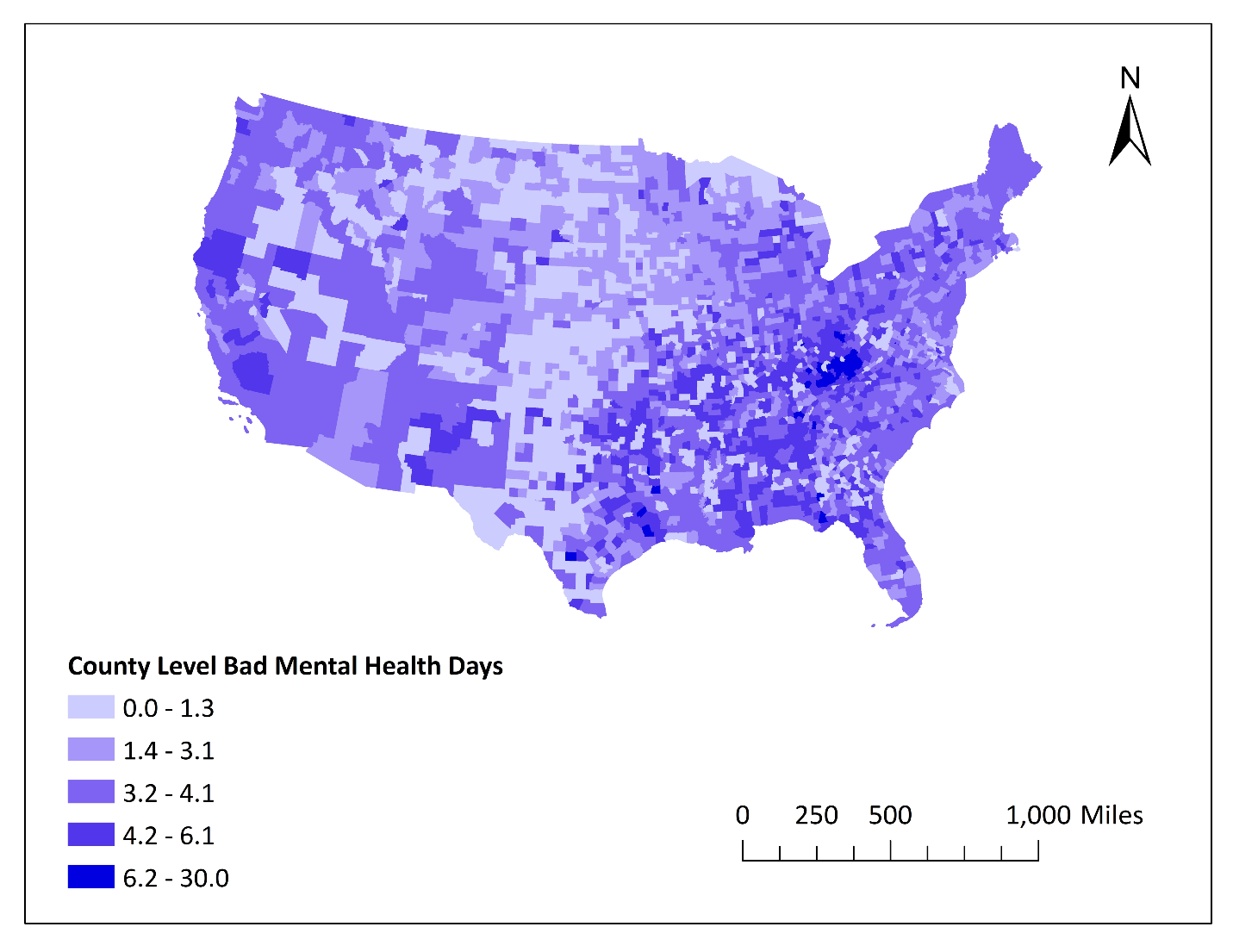


***Notes***: The county-level average number of self-reported bad mental health days is for the period 1993-2010. A darker color signifies more self-reports of bad mental health. The southeastern area near the Gulf of Mexico and areas in Appalachia have higher counts of bad mental days as compared to the rest of the country.
