# Supplementary material for: Temperature and self-reported mental health in the United States
Source: PLoS One. 2020 Mar 25;15(3):e0230316. doi: 10.1371/journal.pone.0230316 (PMC7094821; doi:10.1371/journal.pone.0230316)
Supplement: S1 Table — Marginal effects from logistic regression are displayed. Standard errors are clustered at the county level in parentheses. Controls for State-month, county, day-of-year, and year dummies are also included but not displayed to save space. Survey weight is applied. *** p<0.01, ** p<0.05, * p<0.1. (DOCX) [file pone.0230316.s002.docx]

**S1 Table. Full Regression of Baseline Specification**

|  | (1) |
| --- | --- |
|  | Mental Health Difficulties |
|  |  |
| <20°F | -0.0083^**^ |
|  | (0.0032) |
| 20-30°F | -0.0002 |
|  | (0.0025) |
| 30-40°F | -0.0044^**^ |
|  | (0.0020) |
| 40-50°F | -0.0025^*^ |
|  | (0.0013) |
| 50-60°F | -0.0019^*^ |
|  | (0.0011) |
| 70-80°F | 0.0020^*^ |
|  | (0.0011) |
| ≥80°F | 0.0032^**^ |
|  | (0.0015) |
| Precipitation | 0.0616 |
|  | (0.0567) |
| Sunlight | -0.00002*** |
|  | (0.0000) |
| Dewpoint Temperature | -0.0008 |
|  | (0.0020) |
| Log(income) | -0.1130^***^ |
|  | (0.0114) |
| Age | -0.0033^***^ |
|  | (0.0010) |
| Age squared | -0.0002^***^ |
|  | (0.0000) |
| *(Married as Reference)* |  |
| Divorced | 0.2917^***^ |
|  | (0.0073) |
| Widowed | 0.1767^***^ |
|  | (0.0129) |
| Separated | 0.4642^***^ |
|  | (0.0168) |
| Never married | 0.2015^***^ |
|  | (0.0107) |
| A member of an unmarried couple | 0.3412^***^ |
|  | (0.0163) |
| Children in household | -0.0040 |
|  | (0.0032) |
| ***(Never attended school as Reference)*** | |
| Elementary | -0.0833 |
|  | (0.0744) |
| Some high school | 0.0579 |
|  | (0.0759) |
| High school graduate | -0.0159 |
|  | (0.0717) |
| Some college | 0.0990 |
|  | (0.0716) |
| College graduate | 0.0098 |
|  | (0.0717) |
| ***(Employed as Reference)*** |  |
| Self-employed | 0.0053 |
|  | (0.0109) |
| Out of work > 1 year | 0.4375^***^ |
|  | (0.0185) |
| Out of work < 1 year | 0.3547^***^ |
|  | (0.0161) |
| Homemaker | -0.0317^***^ |
|  | (0.0096) |
| Student | 0.1835^***^ |
|  | (0.0168) |
| Retired | -0.0454^***^ |
|  | (0.0132) |
| Unable to work | 1.1281^***^ |
|  | (0.0171) |
| ***(Male as Reference)*** |  |
| Female | 0.4977^***^ |
|  | (0.0088) |
| ***(With health plan as Reference)*** | |
| No health plan | 0.0606^***^ |
|  | (0.0103) |
| N | 3,055,939 |

***Notes***: Marginal effects from logistic regression are displayed. Standard errors are clustered at the county level in parenthesis. Controls for State-month, county, day-of-year, and year dummies are also included but not displayed to save space. Survey weight is applied. *** p<0.01, ** p<0.05, * p<0.1.
