# Supplementary material for: Temperature and self-reported mental health in the United States
Source: PLoS One. 2020 Mar 25;15(3):e0230316. doi: 10.1371/journal.pone.0230316 (PMC7094821; doi:10.1371/journal.pone.0230316)
Supplement: S2 Table — Columns in Panel A look at the effect of temperature for previous 7 to 30 days accordingly by week, conditional on previous weeks; columns in Panel B look at the temperature effect for previous weeks by week, unconditional on previous weeks. Each column follows the baseline specification in Eq (1). Survey weight is applied, and marginal effects from logistic regressions are displayed. *** p<0.01, ** p<0.05, * p<0.1. (DOCX) [file pone.0230316.s003.docx]

**S2 Table. Effect of Temperature from Previous Days by Week: Conditional and Unconditional**

| **Panel A** | **(1)** | **(2)** | **(3)** | **(4)** |
| --- | --- | --- | --- | --- |
|  | **Previous 7-day** | **Previous 14-day** | **Previous 21-day** | **Previous 30-day** |
| ***Previous 7 days*** |  |  |  |  |
| <20°F | -0.0237^***^ | -0.0240^***^ | -0.0246^***^ | -0.0228^***^ |
|  | (0.0079) | (0.0076) | (0.0071) | (0.0068) |
| 20-30°F | -0.0026 | -0.0027 | -0.0032 | -0.0020 |
|  | (0.0053) | (0.0046) | (0.0042) | (0.0038) |
| 30-40°F | -0.0066 | -0.0062^*^ | -0.0068^**^ | -0.0058^*^ |
|  | (0.0040) | (0.0037) | (0.0034) | (0.0032) |
| 40-50°F | -0.0063^**^ | -0.0061^**^ | -0.0065^**^ | -0.0059^**^ |
|  | (0.0031) | (0.0028) | (0.0027) | (0.0026) |
| 50-60°F | -0.0027 | -0.0022 | -0.0024 | -0.0021 |
|  | (0.0024) | (0.0023) | (0.0023) | (0.0023) |
| 70-80°F | 0.0054^*^ | 0.0039 | 0.0035 | 0.0034 |
|  | (0.0028) | (0.0027) | (0.0026) | (0.0025) |
| ≥80°F | 0.0026 | -0.0015 | -0.0020 | -0.0028 |
|  | (0.0036) | (0.0037) | (0.0035) | (0.0034) |
| ***Previous 8-14 days*** |  |  |  |  |
| <20°F |  | -0.0061 | -0.0095 | -0.0081 |
|  |  | (0.0070) | (0.0067) | (0.0061) |
| 20-30°F |  | -0.0049 | -0.0090^*^ | -0.0079^*^ |
|  |  | (0.0051) | (0.0051) | (0.0047) |
| 30-40°F |  | -0.0090^**^ | -0.0119^***^ | -0.0110^***^ |
|  |  | (0.0040) | (0.0040) | (0.0037) |
| 40-50°F |  | -0.0042 | -0.0064^*^ | -0.0058^*^ |
|  |  | (0.0034) | (0.0037) | (0.0034) |
| 50-60°F |  | -0.0062^***^ | -0.0078^***^ | -0.0075^***^ |
|  |  | (0.0024) | (0.0025) | (0.0024) |
| 70-80°F |  | 0.0042^*^ | 0.0039^*^ | 0.0039^*^ |
|  |  | (0.0023) | (0.0023) | (0.0023) |
| ≥80°F |  | 0.0120^***^ | 0.0111^**^ | 0.0106^**^ |
|  |  | (0.0043) | (0.0046) | (0.0046) |
| ***Previous 15-21 days*** |  |  |  |  |
| <20°F |  |  | 0.0011 | 0.0018 |
|  |  |  | (0.0048) | (0.0049) |
| 20-30°F |  |  | 0.0091** | 0.0086** |
|  |  |  | (0.0042) | (0.0039) |
| 30-40°F |  |  | 0.0021 | 0.0019 |
|  |  |  | (0.0038) | (0.0038) |
| 40-50°F |  |  | 0.0026 | 0.0022 |
|  |  |  | (0.0029) | (0.0030) |
| 50-60°F |  |  | 0.0029 | 0.0023 |
|  |  |  | (0.0022) | (0.0022) |
| 70-80°F |  |  | 0.0034 | 0.0045* |
|  |  |  | (0.0023) | (0.0025) |
| ≥80°F |  |  | 0.0047 | 0.0047 |
|  |  |  | (0.0038) | (0.0039) |
| ***Previous 22-30 days*** |  |  |  |  |
| <20°F |  |  |  | -0.0054 |
|  |  |  |  | (0.0055) |
| 20-30°F |  |  |  | 0.0001 |
|  |  |  |  | (0.0040) |
| 30-40°F |  |  |  | -0.0031 |
|  |  |  |  | (0.0036) |
| 40-50°F |  |  |  | -0.0013 |
|  |  |  |  | (0.0027) |
| 50-60°F |  |  |  | -0.0004 |
|  |  |  |  | (0.0021) |
| 70-80°F |  |  |  | -0.0026 |
|  |  |  |  | (0.0026) |
| ≥80°F |  |  |  | 0.0012 |
|  |  |  |  | (0.0031) |
| N | 3,421,776 | 3,421,776 | 3,421,776 | 3,421,776 |
|  | **(5)** | **(6)** | **(7)** | **(8)** |
| **Panel B** | **Previous Days**  **0-7** | **Previous Days**  **8-14** | **Previous Days 15-21** | **Previous Days 22-30** |
| <20°F | -0.0237^***^ | -0.0076 | 0.0043 | -0.0018 |
|  | (0.0079) | (0.0068) | (0.0048) | (0.0053) |
| 20-30°F | -0.0026 | -0.0032 | 0.0108^**^ | 0.0040 |
|  | (0.0053) | (0.0049) | (0.0042) | (0.0039) |
| 30-40°F | -0.0066 | -0.0075^**^ | 0.0028 | -0.0008 |
|  | (0.0040) | (0.0037) | (0.0037) | (0.0035) |
| 40-50°F | -0.0063^**^ | -0.0041 | 0.0024 | -0.0003 |
|  | (0.0031) | (0.0032) | (0.0028) | (0.0024) |
| 50-60°F | -0.0027 | -0.0064^***^ | 0.0016 | -0.0003 |
|  | (0.0024) | (0.0024) | (0.0022) | (0.0020) |
| 70-80°F | 0.0054^*^ | 0.0040^*^ | 0.0040^*^ | -0.0011 |
|  | (0.0028) | (0.0024) | (0.0023) | (0.0024) |
| ≥80°F | 0.0026 | 0.0087^**^ | 0.0056 | 0.0028 |
|  | (0.0036) | (0.0041) | (0.0036) | (0.0028) |
| N | 3,060,158 | 3,060,158 | 3,060,158 | 3,060,158 |

***Notes***: Columns in Panel A look at the impact of temperature for previous 7 to 30 days accordingly by week, conditional on previous weeks; columns in Panel B look at the temperature impact for previous weeks by week, unconditional on previous weeks. Each column follows the baseline specification in equation (1). Survey weight is applied, and marginal effects from logistic regressions are displayed. *** p<0.01, ** p<0.05, * p<0.1.
