# Supplementary material for: Temperature and self-reported mental health in the United States
Source: PLoS One. 2020 Mar 25;15(3):e0230316. doi: 10.1371/journal.pone.0230316 (PMC7094821; doi:10.1371/journal.pone.0230316)
Supplement: S3 Table — The regression results of marginal effects of the first two columns are supplement for Fig 4; *** p<0.01, ** p<0.05, * p<0.1. (DOCX) [file pone.0230316.s004.docx]

**S3 Table. Effect of Temperature on People with Frequent Mental Distress (FMD)**

|  |  |  |  |
| --- | --- | --- | --- |
|  | **No FMD** | **FMD** | **FMD**  **(Day=30)** |
| <20°F | -0.0008 | -0.0198^***^ | -0.0323^***^ |
|  | (0.0024) | (0.0063) | (0.0084) |
| 20-30°F | 0.0037^**^ | -0.0076 | -0.0093 |
|  | (0.0018) | (0.0050) | (0.0066) |
| 30-40°F | -0.0018 | -0.0078^*^ | -0.0115^**^ |
|  | (0.0016) | (0.0040) | (0.0054) |
| 40-50°F | -0.0001 | -0.0067^**^ | -0.0073^**^ |
|  | (0.0012) | (0.0026) | (0.0035) |
| 50-60°F | 0.0006 | -0.0057^**^ | -0.0116^***^ |
|  | (0.0013) | (0.0023) | (0.0033) |
| 70-80°F | 0.0002 | 0.0052^***^ | 0.0059^*^ |
|  | (0.0009) | (0.0019) | (0.0030) |
| ≥80°F | 0.0003 | 0.0081^***^ | 0.0103^**^ |
|  | (0.0011) | (0.0024) | (0.0041) |
| *N* | 2,620,075 | 2,380,823 | 2,227,604 |

***Notes*:** The regression results of marginal effects of the first two columns are supplement for Fig 4; *** p<0.01, ** p<0.05, * p<0.1.
