# Supplementary material for: Temperature and self-reported mental health in the United States
Source: PLoS One. 2020 Mar 25;15(3):e0230316. doi: 10.1371/journal.pone.0230316 (PMC7094821; doi:10.1371/journal.pone.0230316)
Supplement: S1 Text — Derive the marginal effect from the logistic regression. (DOCX) [file pone.0230316.s005.docx]

**S1 Text. Marginal Effect Calculation**

Given our empirical setting of the logistic regression in equation (1), we simplify the function to include one temperature bin and the log of annual household income while putting everything else to W for the convenience of illustration. The simplified function is:

$P\left( z \right)=\frac{\exp(z)}{1+exp(z)} , where z=\alpha+ \beta_{1}Tbin+ \beta_{2}ln(Inc)+W$. (3)

Notice that the first order derivative to $Tbin$ is $z^{'}= \beta_{1}$ and the first order derivative to z in P(z) is:

$$P^{'}\left( z \right)=\exp\left( z \right)\left[ 1+\exp\left( z \right) \right]^{-1}+{\exp\left( z \right)}^{2}\left( -1 \right)\left[ 1+\exp\left( z \right) \right]^{-2}$$

$=\frac{exp(z)}{{[1+\exp\left( z \right)]}^{2}}$

$=P\left( z \right)[1-P\left( z \right)]$. (4)

Thus, the marginal effect of an additional day with extreme temperature in bin on the probability of self-reported mental health difficulties is:

$\frac{\partial P\left( z \right)}{\partial Tbin}= \beta_{1} P\left( z \right)\left[ 1-P\left( z \right) \right].$ (5)

Similarly, the marginal effect of income is:

$\frac{\partial P\left( z \right)}{\partial Inc}=\left( -\frac{1}{\bar{Inc}}\beta_{2} \right)P\left( z \right)\left[ 1-P\left( z \right) \right]$.

$= -\frac{1}{\bar{Inc}} \frac{\partial P\left( z \right)}{\partial ln(Inc)}$ . (6)

Therefore, the average marginal rate of substitution between temperature and annual household income holding the probability of self-reported mental health difficulties constant is:

$\frac{\partial Inc}{\partial Tbin}|_{\partial P\left( z \right)=0}=-\bar{Inc}(\frac{\partial P\left( z \right)}{\partial Tbin}/\frac{\partial P\left( z \right)}{\partial ln(Inc)})$. (7)
